# Misplaced Charity

**Published:** 1914-02

**Authors:** 


### MISPLACED CHARITY

Recent proceedings against the branch of the Volunteers of America, in Buffalo, have shown a very child-like method of deducting maintenance charges from contributions and incompetence in the management of some philanthropies. It ought to have been self-evident that a class of the population that normally would be self-supporting only by simple forms of labor, cannot lead the life of a clergy educated for religious instruction, travel extensively, and wear very good clothes, without the support from the gifts originally intended for the disabled poor and those temporarily out of work. In view of the large number of persons supporting themselves by "army" work, it is obvious that the economic loss must be considerable. It is a good plan to limit charity to individuals personally known to be deserving and to institutions conducted by a class which is beyond the need of charity and therefore, beyond the temptation, either ignorantly or as a matter of graft, to appropriate from such funds for its own support. And, granting that there are sufficient institutions for the care of the obviously defective members of society, a request for charity usually means drunkenness, laziness or at least an unwillingness to work except at high wages, and shiftlessness. Barring great and repeated misfortunes, the deserving poor who apply for or accept charity, are very few, at least if we consider the family as a unit. Of course, individual dependent members may be deserving, though their needs arise from the fact that the normal bread winner is undeserving.

